# Editorial: Genetics of Familial Hypercholesterolemia: New Insight

**DOI:** 10.3389/fgene.2021.669373

**Published:** 2021-05-06

**Authors:** Alpo Vuorio, Uma Ramaswami, Kirsten B. Holven

**Affiliations:** ^1^Mehiläinen Airport Health Centre, Vantaa, Finland; ^2^Department of Forensic Medicine, University of Helsinki, Helsinki, Finland; ^3^Lysosomal Disorders Unit, Royal Free London National Health Service Foundation Trust, London, United Kingdom; ^4^Genetics and Genomics Department, University College London, London, United Kingdom; ^5^Department of Nutrition, Institute of Basic Medical Sciences, University of Oslo, Oslo, Norway; ^6^Norwegian National Advisory Unit on Familial Hypercholesterolemia, Department of Endocrinology, Morbid Obesity and Preventive Medicine, Oslo University Hospital, Oslo, Norway

**Keywords:** familial hypercholesterolemia, mutations, atherosclerosis, LDL cholesterol, statins, PCSK9 inhibitors

The Research Topic “Genetics Familial Hypercholesterolemia: New Insight” attracted over 100 authors from 16 countries to submit manuscripts on topical genetic research in familial hypercholesterolemia (FH). Encouragingly, over fifty percent of the manuscripts were from low- and middle-income countries researchers from very different backgrounds. This achieved our goal of bringing together researchers from worldwide. It also reaffirmed conclusions from Vrablik et al. review article: “The coordinated international efforts should increase the chances of achieving the principal goal—to identify, diagnose and provide treatment for all FH patients early enough.” The strength of this review topic was the submission of manuscripts from researchers at different stages of their research portfolio. For example, Dušková et al. analyzed in detail pathogenic variants in the low-density lipoprotein gene and their effects on protein localization, function and expression of genes associate endoplasmic reticulum representing advanced molecular biology approaches. At the same time Vasilyev et al. were working with basic molecular biology questions and they concluded that “major achievements in the genetic investigation of the molecular features of FH in Russia are yet to come.”

Unfortunately, FH still remains vastly underdiagnosed disease. As an example, is the study by Ramaswami et al. (2019) that was carried out in UK pediatric population. This study demonstrated that <550 children with a confirmed diagnosis of heterozygous FH (HeFH) were being managed in the health care system. This was despite an estimated prevalence of 50,000 children under the age of 18 years with HeFH in the UK based on a population prevalence of 1 in 250. Once diagnosed, it is essential to enable long-term follow-up of these patients and collating longitudinal data on the management and safety of disease modifying therapies such as statins and newer emerging treatments. This would be possible with both national and international HeFH registries. These registries could also potentially help genotype-phenotype correlations related to different HeFH pathogenic mutations in the *LDLR, APOB*, or *PCSK9* genes. This could also apply to genes associated to HeFH diagnosis, and which are differentially expressed compared to non-FH controls Udhaya Kumar et al..

Truong et al. described the Vietnam Familial Registry (VINAFH) and reported that the VINAFH registry have had a favorable effect on government legislative policies. This highlighted the importance of registries in managing and providing evidence based supportive information for HeFH populations in low- and middle-income countries, and indeed we believe that national and international registries are a key part of management and follow up of both adults and children with HeFH globally. The advantage of HeFH registries would apply especially to large Asian countries where the HeFH diagnosis rates are low Huang and Charng and where HeFH awareness is also still low amongst health care professionals.

In addition to clinically suspected cases of HeFH who are mutation-negative for *LDLR, APOB*, or PCSK9 mutations, there are patients with severe hypercholesterolemia who have an accumulation of common small-effect LDL cholesterol raising alleles (Talmud et al., 2013). These patients are defined as “polygenic hypercholesterolemia.” Jarauta et al. highlighted the importance of newer genetic methods to diagnose “polygenic hypercholesterolemia” early and to ensure initiation of a lipid lowering therapy in a timely manner. However, the genetic criterion for the diagnosis of “polygenic hypercholesterolemia” remains a challenge. Genetic testing for monogenic HeFH could potentially also help identify “polygenic hypercholesterolemia” but this option is not utilized effectively Kamar et al..

Recently, Futema et al. (2021) demonstrated that both children and adults with HeFH caused by pathogenic *LDLR* mutation have a higher LDL cholesterol concentration compared to HeFH caused by *APOB* mutation.

Healthy diet and lifestyle and early statin treatment are cornerstones in HeFH treatment (Vuorio et al., 2019; Rodríguez-Borjabad et al., 2021). Statin treatment has been shown to be safe and effective in children with HeFH (Humphries et al., 2018; Vuorio et al., 2019). However, many children with HeFH are not on appropriate lipid-lowering treatments (Ramaswami et al., 2020), including our European patients. It has been speculated that the widespread use of genetic testing has been crucial for the increased statin use in Norway, especially in young children with HeFH aged 10–19 years compared to their Scandinavian neighbors with less prevalent genetic testing (Svendsen et al., 2021). Despite this, the prevalence of patients with HeFH among patients with premature acute myocardial infarction (AMI) remains significantly higher compared to the general population in Norway (Bogsrud et al., 2020). The most likely explanation is the increased lifelong cholesterol burden prior to their AMI, and an increased LDL cholesterol concentration at the time of AMI. Similar results were also confirmed by Moradi et al. in Iran. It has been also shown that mortality and the risk of recurrent AMI is increased in HeFH compared with non-FH controls (Svendsen et al., 2020). A poor prognosis after the first AMI in HeFH patients was also confirmed in this Research Topic by Arnesen et al..

PCSK9 inhibitors represent a relatively new class of medication that targets the unique mechanism of action of *PCSK9* Guo et al.. The effectiveness and safety of PCSK9 inhibitor was shown recently in pediatric HeFH (Santos et al., 2020). In this 24-week study, the mean LDL cholesterol lowering was −44.5% and −6.2% in children with HeFH on evolocumab, a PCSK9 inhibitor, and a control group, respectively. Inclisiran (Hovingh et al., 2020) highlights the effective use of such adjunctive therapies that potentially help achieve appropriate target LDL cholesterol concentration and better clinical outcomes. Oommen et al. described that over half of the HeFH causing LDLR mutations resulted in protein misfolding, defective transport and trafficking, with the misfolded proteins being retained in the endoplasmic reticulum (ER). The authors postulate drugs modulating proteostasis in the ER as therapeutic alternatives for patients who have persistently elevated LDL cholesterol despite optimization of conventional lipid lowering therapies.

Homozygous form of FH (HoFH) is a rare disease with a prevalence ~1:200,000–30,000 (Sjouke et al., 2015). HoFH is a serious disease due to very aggressive atherosclerosis progression. Without early interventions, HoFH causes AMI in childhood (Vallejo-Vaz et al., 2015). In the current Research Topic, Marusic et al. showed that HoFH patients represent a clinically heterogenous group. In some cases, the HoFH phenotype may overlap with HeFH, and hence genetic testing is paramount. The authors highlight the lack of therapeutic options for HoFH patients in less developed countries. Mlinaric et al. reported an interesting case of liver transplantation (LT) in a HoFH child with progressive atherosclerosis despite early LDL-apheresis. The authors suggest LT as a feasible option especially in HoFH unresponsive to lipid lowering therapies and/or LDL-apheresis. The authors also suggest the benefits of an international registry for HoFH and LT. The International Registry on Lipoprotein Apheresis in Children with HoFH could serve as an example (Luirink et al., 2020).

The most recent challenge related to FH is the COVID-19 pandemic (Vuorio et al., 2021a). It can be estimated that with a HeFH prevalence of 1 in 250, ~440,000 HeFH patients may have had SARS-Cov-2 infection by February 2021. In severely ill COVID-19 patients, HeFH could potentially be overrepresented due to endothelial dysfunction and atherosclerotic cardiovascular disease as risk factors (Vuorio et al., 2020). A lifelong elevated LDL cholesterol concentration is often associated with an increased lipoprotein(a) Lp(a), with endothelial dysfunction starting in childhood. In a meta-analysis carried among hospitalized COVID-19 patients, AMI was present in 3.3% (95% CI 0.3–8.5) of the cases (Kunutsor and Laukkanen, 2020). A meta-analysis of COVID-19 studies revealed that statin use decreases not only the mortality but also the severity of SARS-Cov-2 infection significantly (OR 0.51, 95% CI 0.41–0.64) (Onorato et al., 2021). Therefore, among patients with HeFH, an effective lowering of LDL cholesterol concentration is essential to improve endothelial dysfunction (Iqbal et al., 2020; Vuorio and Kovanen, 2020) and potentially reduce COVID-19 risk. PSCSK9 inhibitors could also be considered as an adjunctive therapy to effectively lowering both LDL cholesterol concentration and Lp(a), with also potential antiviral properties (Iqbal et al., 2020; Vuorio and Kovanen, 2021). An effective lowering of LDL cholesterol concentration, especially in older patients with HeFH is important to mitigate the higher risk of becoming critically ill with COVID-19 (Vuorio et al., 2021b). Currently, there is a great need to collect comprehensive epidemiologic data with the aid of international collaboration among the centers on the clinical course and outcomes of FH patients who have contracted COVID-19 infection.

## Author Contributions

AV: writing the first draft. AV, UR, and KV: reviewing and editing to produce the final draft. All authors contributed to the article and approved the submitted version.

## Conflict of Interest

KH has received research grants or honoraria from Mills AS, Tine SA, Olympic Seafood, Amgen, Sanofi, none of which are related to the content of this manuscript. The remaining authors declare that the research was conducted in the absence of any commercial or financial relationships that could be construed as a potential conflict of interest.

## References

[B1] Bogsrud M. P. Øyri L. Halvorsen S Atar D. Leren T. P. Holven K. B. (2020). Prevalence of genetically verified familial hypercholesterolemia among young (<45 years) Norwegian patients hospitalized with acute myocardial infarction. J. Clin. Lipidol. 14, 339–345. 10.1016/j.jacl.2020.04.002 32418822

[B2] Futema M. Ramaswami U. Tichy L. Bogsrud M. P. Holven K. B. Roeters van Lennep J.. (2021). Comparison of the mutation spectrum and association with pre and post treatment lipid measures of children with heterozygous familial hypercholesterolaemia (FH) from eight European countries. Atherosclerosis 319, 108–117. 10.1016/j.atherosclerosis.2021.01.008 33508743

[B3] Hovingh G. K. Lepor N. E. Kallend D. Stoekenbroek R. M. Wijngaard P. L. J. Raal F. J. (2020). Inclisiran durably lowers low-density lipoprotein cholesterol and proprotein convertase subtilisin/kexin type 9 expression in homozygous familial hypercholesterolemia: the ORION-2 Pilot study. Circulation 141, 1829–1831. 10.1161/CIRCULATIONAHA.119.044431 32479195

[B4] Humphries S. E. Cooper J. Dale P. Ramaswami U. F. H Paediatric Register Steering Group (2018). The UK Paediatric familial hypercholesterolaemia register: statin-related safety and 1-year growth data. J. Clin. Lipidol. 12, 25–32. 10.1016/j.jacl.2017.11.005 29208363 PMC5821682

[B5] Iqbal Z. Ho J. H. Adam S. France M. Syed A. Neely D. (2020). Managing hyperlipidaemia in patients with COVID-19 and during its pandemic: an expert panel position statement from HEART UK. Atherosclerosis 313, 126–136. 10.1016/j.atherosclerosis.2020.09.008 33045618 PMC7490256

[B6] Kunutsor S. K. Laukkanen J. A. (2020). Incidence of venous and arterial thromboembolic complications in COVID-19: a systematic review and meta-analysis. Thromb. Res. 196, 27–30. 10.1016/j.thromres.2020.08.022 32823173 PMC7418701

[B7] Luirink I. K. Hutten B. A. Greber-Platzer S. Kolovou G. D. Dann E. J. de Ferranti S. D.. (2020). Practice of lipoprotein apheresis and short-term efficacy in children with homozygous familial hypercholesterolemia: data from an international registry. Atherosclerosis 299, 24–31. 10.1016/j.atherosclerosis.2020.01.031 32199148

[B8] Onorato D. Pucci M. Carpene G. Henry B. M. Sanchis-Gomar F. Lippi G. (2021). Protective effects of statins administration in European and North American patients infected with COVID-19: a meta-analysis. Sem. Thromb. Hemos. 10.1055/s-0040-1722307 33482680

[B9] Ramaswami U. Futema M. Bogsrud M. P. Holven K. B. Roeters van Lennep J. Wiegman A.. (2020). Comparison of the characteristics at diagnosis and treatment of children with heterozygous familial hypercholesterolaemia (FH) from eight European countries. Atherosclerosis 292, 178–187. 10.1016/j.atherosclerosis.2019.11.012 31809987 PMC6949888

[B10] Ramaswami U. Humphries S. E. Priestley-Barnham L. Green P. Wald D. S. Capps N.. (2019). Current management of children and young people with heterozygous familial hypercholesterolaemia - HEART UK statement of care. Atherosclerosis 290, 1–8. 10.1016/j.atherosclerosis.2019.09.005 31536851

[B11] Rodríguez-Borjabad C. Narveud I. Christensen J. J. Ulven S. M. Malo A. I. Ibarretxe D.. (2021). Dietary intake and lipid levels in Norwegian and Spanish children with familial hypercholesterolemia. Nutr. Metab. Cardiovasc. Dis. 31, 1299–1307. 10.1016/j.numecd.2020.12.002 33549456

[B12] Santos R. D. Ruzza A. Hovingh G. K. Wiegman A. Mach F. Kurtz C. E.. (2020). Evolocumab in pediatric heterozygous familial hypercholesterolemia. NEJM 383, 1317–1327. 10.1056/NEJMoa2019910 32865373

[B13] Sjouke B. Hovingh G. K. Kastelein J. J. Stefanutti C. (2015). Homozygous autosomal dominant hypercholesterolaemia: prevalence, diagnosis, and current and future treatment perspectives. Curr. Opin. Lipidol. 26, 200–209. 10.1097/MOL.0000000000000179 25950706

[B14] Svendsen K. Krogh H. W. Igland J. Tell G. S. Mundal L. J. Holven K. B.. (2020). 2.5-fold increased risk of recurrent acute myocardial infarction with familial hypercholesterolemia. Atherosclerosis 319, 28–34. 10.1016/j.atherosclerosis.2020.12.019 33465659

[B15] Svendsen K. Langslet G. Krogh H. W. Brinck J. Klausen I. C. Stenehjem J. S.. (2021). Genetic testing is essential for initiating statin therapy in children with familial hypercholesterolemia: examples from Scandinavia. Atherosclerosis 316, 48–52. 10.1016/j.atherosclerosis.2020.11.027 33302044

[B16] Talmud P. J. Shah S. Whittall R. Futema M. Howard P. Cooper J. A.. (2013). Use of low-density lipoprotein cholesterol gene score to distinguish patients with polygenic and monogenic familial hypercholesterolaemia: a case-control study. Lancet 381, 1293–1301. 10.1016/S0140-6736(12)62127-8 23433573

[B17] Vallejo-Vaz A. J. Kondapally Seshasai S. R. Cole D. Hovingh G. K. Kastelein J. J. Mata P.. (2015). Familial hypercholesterolaemia: a global call to arms. Atherosclerosis 243, 257–259. 10.1016/j.atherosclerosis.2015.09.021 26408930

[B18] Vuorio A. Kovanen P. T. (2020). Prevention of endothelial dysfunction and thrombotic events in COVID-19 patients with familial hypercholesterolemia. J. Clin. Lipidol. 14, 617–618. 10.1016/j.jacl.2020.06.006 32653497 PMC7295490

[B19] Vuorio A. Kovanen P. T. (2021). PCSK9 inhibitors for COVID-19: an opportunity to enhance the antiviral action of interferon in patients with hypercholesterolaemia. J. Int. Med. 289, 749–751. 10.1111/joim.13210 33222314 PMC7753347

[B20] Vuorio A. Kovanen P. T. Strandberg T. E. (2021b). Older familial hypercholesterolemia patients with COVID-19. Gerontology 18, 1–3. 10.1159/000514447 33735871

[B21] Vuorio A. Kuoppala J. Kovanen P. T. Humphries S. E. Tonstad S. Wiegman A.. (2019). Statins for children with familial hypercholesterolemia. Cochrane Database Syst. Rev. 2019:CD006401. 10.1002/14651858.CD006401.pub5 PMC6836374 31696945

[B22] Vuorio A. Raal F. Kaste M. Kovanen P. T. (2021a). Familial hypercholesterolaemia and COVID-19: a two-hit scenario for endothelial dysfunction amenable to treatment. Atherosclerosis 320, 53–60. 10.1016/j.atherosclerosis.2021.01.021 33540179 PMC7830285

[B23] Vuorio A. Watts G. F. Kovanen P. T. (2020). Familial hypercholesterolaemia and COVID-19: triggering of increased sustained cardiovascular risk. J. Int. Med. 287, 746–747. 10.1111/joim.13070 32242993

